# Engineering MoS_2_ Basal Planes for Hydrogen Evolution via Synergistic Ruthenium Doping and Nanocarbon Hybridization

**DOI:** 10.1002/advs.201900090

**Published:** 2019-03-20

**Authors:** Xing Zhang, Feng Zhou, Shen Zhang, Yongye Liang, Ruihu Wang

**Affiliations:** ^1^ State Key Laboratory of Structural Chemistry Fujian Institute of Research on the Structure of Matter Chinese Academy of Sciences Fuzhou Fujian 350002 China; ^2^ Department of Materials Science and Engineering Southern University of Science and Technology Shenzhen 518055 China; ^3^ University of Chinese Academy of Sciences Beijing 100049 China

**Keywords:** carbon nanotubes, core/shell structure, hydrogen evolution electrocatalysis, molybdenum sulfide, ruthenium doping

## Abstract

Promoting the intrinsic activity and accessibility of basal plane sites in 2D layered metal dichalcogenides is desirable to optimize their catalytic performance for energy conversion and storage. Herein, a core/shell structured hybrid catalyst, which features few‐layered ruthenium (Ru)‐doped molybdenum disulfide (MoS_2_) nanosheets closely sheathing around multiwalled carbon nanotube (CNT), for highly efficient hydrogen evolution reaction (HER) is reported. With 5 at% (atomic percent) Ru substituting for Mo in MoS_2_, Ru‐MoS_2_/CNT achieves the optimum HER activity, which displays a small overpotential of 50 mV at −10 mA cm^−2^ and a low Tafel slope of 62 mV dec^−1^ in 1 m KOH. Theoretical simulations reveal that Ru substituting for Mo in coordination with six S atoms is thermodynamically stable, and the in‐plane S atoms neighboring Ru dopants represent new active centers for facilitating water adsorption, dissociation, and hydrogen adsorption/desorption. This work provides a multiscale structural and electronic engineering strategy for synergistically enhancing the HER activity of transition metal dichalcogenides.

Hydrogen production by water electrolysis is a centralized energy technology for indirect conversion and storage of sustainable but intermittent energy sources, such as solar energy, wind energy, and tide energy.^1^, ^2^ Due to high intrinsic catalytic activity, excellent chemical stability, and earth abundance, 2D layered transition metal dichalcogenides (e.g., MoS_2_ and WS_2_) have been regarded as the most promising candidates in substituting noble‐metal platinum for catalyzing the cathodic hydrogen evolution reaction (HER) in water splitting.^3^, ^4^, ^5^ Recently, theoretical and experimental studies have demonstrated that only the coordinatively unsaturated sulfur (S) and molybdenum (Mo) atoms along the edges of S‐Mo‐S mole‐cular layers in semiconducting 2H‐MoS_2_ are the catalytically active sites, while a significant proportion of S atoms in 2H‐MoS_2_ basal planes are inert.^6^, ^7^ Although extensive efforts have been paid to maximally expose the active edge sites of 2H‐MoS_2_ for promoting the HER catalytic activities of MoS_2_‐based catalysts,^8^, ^9^, ^10^ it is still a daunting challenge to realize extremely high density of active sites in 2H‐MoS_2_ due to inherently high surface energy of the edges and instabilities of the coordinatively unsaturated edge atoms.^9^, ^11^ Metallic 1T‐MoS_2_ has recently been proposed to display excellent HER activity due to its superior HER energetics on the basal plane.^11^, ^12^, ^13^, ^14^ Nevertheless, the metastable nature of 1T‐MoS_2_ poses a vital concern for long‐term operational stability in practical applications.^15^, ^16^ Therefore, it holds great significance to develop effective strategies for triggering the catalytic ability of inert 2H‐MoS_2_ basal planes.

Heteroatom doping has been identified to be a promising route in enhancing the intrinsic activities of pristine active sites and/or triggering new active centers in MoS_2_‐based HER catalysts.^17^, ^18^, ^19^, ^20^ It has been predicted that doping transition metal atoms (Fe, Co, Ni, and Cu) into 2H‐MoS_2_ edges can enhance HER catalytic activity of S‐edge sites. Unfortunately, the experimental results have demonstrated that the edge doping has very weak influences on the apparent HER activities of doped and edge‐terminated MoS_2_ catalysts, which is possibly attributed to the decrement of intrinsic activities of Mo‐edge sites.^21^ Some recent reports have theoretically demonstrated that the incorporation of dopant atoms (e.g., Pt, Pd, Co, and Ni) can trigger HER catalytic activity in the inert 2H‐MoS_2_ basal planes, while the effects of the doping atoms in the edges and basal planes on HER activity of free MoS_2_ nanosheets are hardly distinguished experimentally.^22^, ^23^, ^24^, ^25^, ^26^ Besides, poor conductivity and restacking of MoS_2_ nanosheets are two important factors, which depress HER catalytic activity in reported MoS_2_‐based electrocatalysts.^27^, ^28^, ^29^, ^30^ Taken together, multiscale electronic and structural engineering would be more effective to upgrade apparent HER activities of MoS_2_‐based catalysts by synergistically enhancing the intrinsic activity of each active site and raising the density of electrochemically accessible active sites.

In this work, we systematically investigated the effects of ruthenium (Ru) doping into the basal plane of 2H‐MoS_2_ on its HER activity. Experimentally, we prepared composition‐tunable Ru‐doped MoS_2_ nanosheets, which epitaxially sheath around multiwalled carbon nanotube (CNT). This unique core/shell structure of Ru‐MoS_2_/CNT can ensure fast charge transfer, efficient mass transport, and exclusive exposure of the basal plane atoms of Ru‐MoS_2_. Electrochemical characterizations indicate that Ru‐doping significantly promotes HER catalytic activities of Ru‐MoS_2_/CNT in terms of exchange current density, onset potential, and Tafel slope. Theoretically, density functional theory (DFT) calculations reveal that Ru‐doping in MoS_2_ effectively modulates the electronic properties of the adjacent in‐plane S atoms, which displays optimum hydrogen binding energy and significantly reduced energy barriers for water adsorption and dissociation.

Ru‐MoS_2_/CNT was prepared according to our previously reported method for the synthesis of MoS_2_/CNT with the introduction of RuCl_3_ as the dopant source (see experimental details in the Supporting Information).^31^ The detailed structural features of representative Ru‐MoS_2_/CNT with Ru doping amount of 5 at% were first investigated by transmission electron microscopy (TEM) and X‐ray diffraction (XRD). Except for a weak diffraction signal around 26.2° from CNT, other diffraction peaks in the XRD pattern of 5%Ru‐MoS_2_/CNT are indexed to hexagonal 2H‐MoS_2_ (Figure S1, Supporting Information). Z‐contrast scanning TEM (STEM) and bright‐field TEM images indicate that the microstructural units of 5%Ru‐MoS_2_/CNT feature hollow tubular structure with smooth surface (**Figure** **1**a,b). High‐resolution TEM images show that few‐layered MoS_2_ nanosheets epitaxially sheath around the CNT core (Figure 1c and Figure S2, Supporting Information). Energy‐dispersive X‐ray spectroscopy (EDS) elemental mapping results confirm highly uniform distribution of Mo, S, and Ru elements in the Ru‐MoS_2_ shell around CNT (Figure 1d). It is noted that cubic phase RuS_2_ nanocrystals (≈3–8 nm in size) were intimately grown on CNT when MoCl_5_ was completely replaced by RuCl_3_ in the synthesis of Ru‐MoS_2_/CNT (Figure S3, Supporting Information). However, there are no diffraction peaks assignable to RuS_2_ in the XRD pattern of Ru‐MoS_2_/CNT with Ru doping amount up to 10 at% (Figure S4 and Table S1, Supporting Information). Because the atomic number of Ru is very close to that of Mo and the Ru‐doping amount is relatively low, no obvious peak shifts are observed in the XRD patterns of Ru‐MoS_2_/CNT. TEM characterizations further confirm the absence of observable crystalline RuS_2_ species in 10%Ru‐MoS_2_/CNT (Figure S5, Supporting Information). Furthermore, our theoretical calculation results have demonstrated that Ru atoms could be stably doped into 2H‐MoS_2_ by substituting Mo atoms and coordinating to six S atoms (Figure S6, Supporting Information). In light of above results, it could be firmly validated that Ru atoms are uniformly doped into the MoS_2_ shell of Ru‐MoS_2_/CNT (Figure 1e).

**Figure 1:**
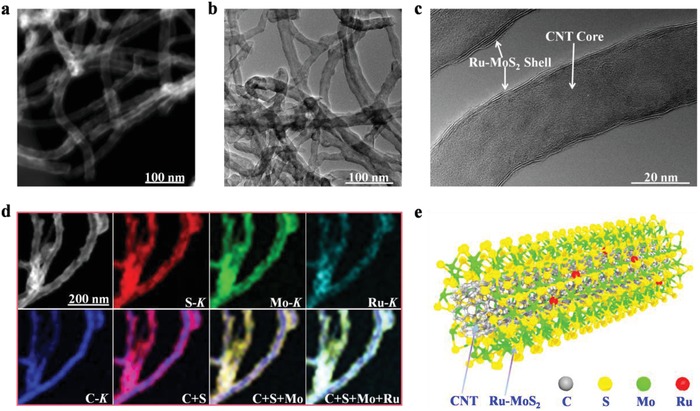
Structural characterizations of 5%Ru‐MoS_2_/CNT. a) STEM, b,c) TEM images, and d) STEM‐EDS elemental mappings. e) Simplified schematic illustration of Ru‐MoS_2_/CNT microstructure.

Multiple spectroscopic characterizations were performed to investigate the structural and electronic properties of Ru‐MoS_2_/CNT. Raman spectra of CNT, MoS_2_/CNT, and 5%Ru‐MoS_2_/CNT were acquired to probe the effects of Ru‐doping on the crystalline structure of MoS_2_ and the interactions between the CNT core and Ru‐MoS_2_ shell (**Figure** **2**a). Both MoS_2_/CNT and 5%Ru‐MoS_2_/CNT exhibit two characteristic in‐plane (E^1^_2g_) and out‐of‐plane (A_1g_) vibration peaks of 2H‐MoS_2_. The inappreciable shift and broadening of E^1^_2g_ and A_1g_ peaks as well as similar intensity ratio of E^1^_2g_/A_1g_ indicate that Ru‐doping does not introduce substantial structural defects or lattice strain in MoS_2_.^32^ In the high‐frequency region, the Raman spectra of these three samples exhibit two distinct peaks centered at 1348 and 1590 cm^−1^, which are associated with the D and G vibration modes of graphitic structure, respectively.^33^ Notably, the intensity ratios of D/G in Raman spectra of MoS_2_/CNT and 5%Ru‐MoS_2_/CNT are reduced to 0.76 and 0.73, respectively, significantly smaller than that of CNT (1.07), suggesting partial removal of oxygen‐containing defects on CNT (Figure S7, Supporting Information). The decrease of defects on CNT is due to the removal of oxygen‐containing groups and reordering of graphitic basal planes during thermal sulfidation in the preparation of Ru‐MoS_2_/CNT.^34^ The less defective CNT core could endow higher charge mobility in Ru‐MoS_2_/CNT. Additionally, the disappearance of 2D vibration peaks in the Raman spectra of MoS_2_/CNT and 5%Ru‐MoS_2_/CNT is probably caused by the strains at the interfaces between CNT core and MoS_2_ or Ru‐MoS_2_ shells, indicating a strong interface interaction that is favorable to the charge transfer.^34^, ^35^, ^36^

**Figure 2:**
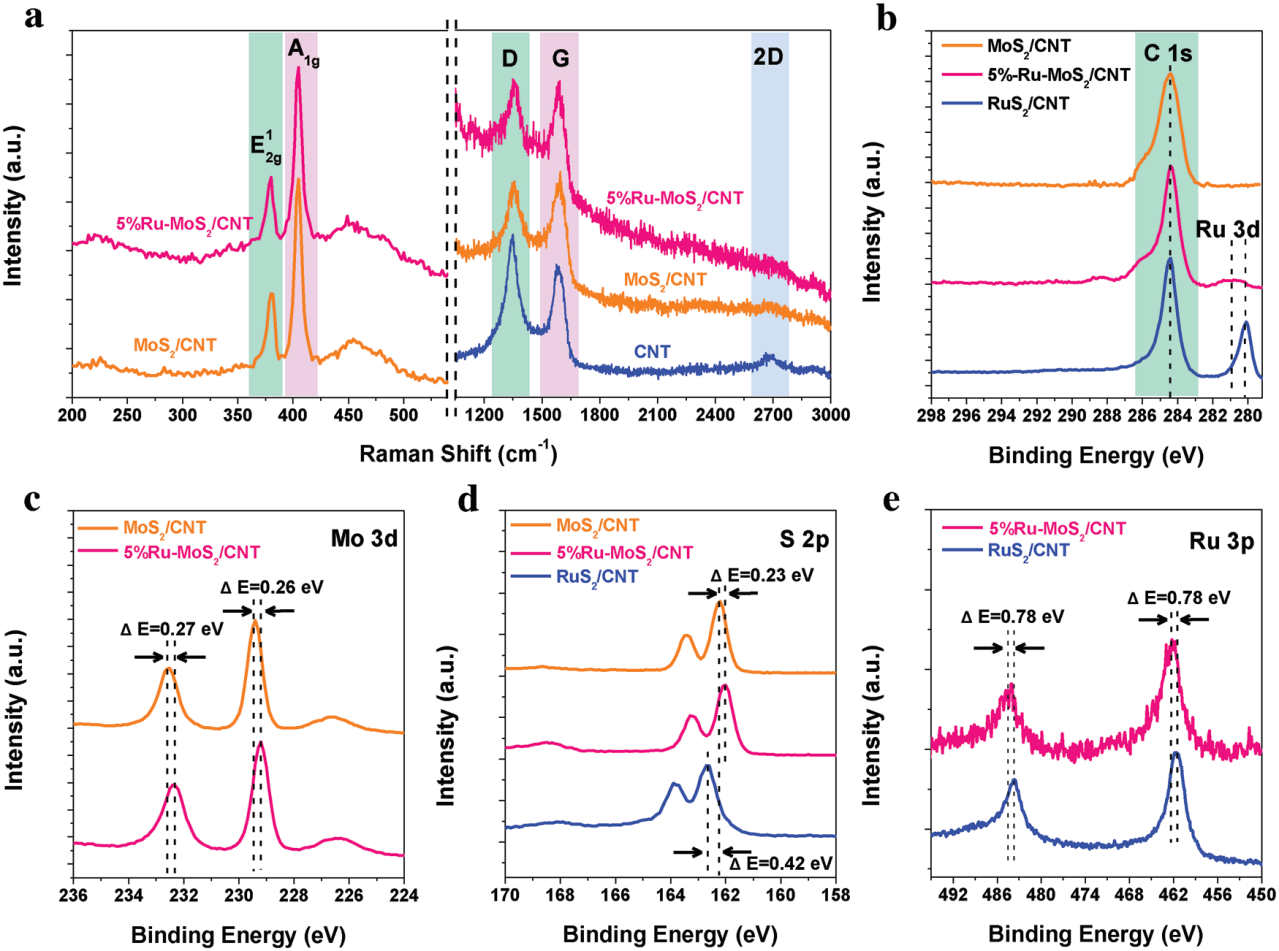
Spectroscopical characterizations of 5%Ru‐MoS_2_/CNT. a) Raman spectra, b–e) high‐resolution XPS spectra of C 1s, Mo 3d, S 2p, and Ru 3p core levels, respectively.

X‐ray photoelectron spectroscopy (XPS) analyses were further performed to study the electronic effects on Ru‐MoS_2_/CNT induced by Ru‐doping. All the adventitious carbon peaks in XPS spectra of the surveyed samples were first calibrated to 284.5 eV (Figure 2b). Compared to those of MoS_2_/CNT, both Mo 3d and S 2p XPS spectra of 5%Ru‐MoS_2_/CNT show a shift of 0.25 (±0.02) eV toward low binding energy, which is probably attributed to the combined effect of the work function change and altered electronic structures of Mo and S in Ru‐MoS_2_/CNT (Figure 2c,d).^37^, ^38^ The negative shifts of Mo 3d and S 2p spectra are consistently observed in Ru‐MoS_2_/CNT with different Ru‐doping amount (Figure S8, Supporting Information). Notably, the binding energy peaks of S 2p and Ru 3p core levels in RuS_2_/CNT are significantly different from those in Ru‐MoS_2_/CNT, displaying a positive shift of 0.65 eV and a negative shift of 0.78 eV, respectively (Figure 2d,e). The energy shift is also observed in the Ru 3d XPS spectra (Figure 2b). These results not only indicate that the chemical states of Ru atoms in Ru‐MoS_2_/CNT are significantly different from those in RuS_2_/CNT but also provide a powerful evidence that Ru atoms are stably doped into MoS_2_ lattice.

Alkaline HER is more competitive to be enrolled in large‐scale hydrogen production in viewing that very few low‐cost electrocatalysts possess satisfactory activities and stabilities for anodic oxygen evolution reaction in acidic or neutral media.^39^, ^40^, ^41^, ^42^, ^43^, ^44^, ^45^, ^46^, ^47^, ^48^, ^49^, ^50^, ^51^, ^52^, ^53^ Herein, the electrocatalytic HER performance of Ru‐MoS_2_/CNT hybrids was evaluated in alkaline 1 m KOH electrolyte. The polarization curves in **Figure** **3**a show that the HER activities of Ru‐MoS_2_/CNT are significantly higher than that of MoS_2_/CNT and sensitive to Ru doping amount. 5%Ru‐MoS_2_/CNT exhibits the highest electrocatalytic activity among these Ru‐MoS_2_/CNT hybrids and is even better than RuS_2_/CNT (Figure S9, Supporting Information). Specifically, for achieving *j* = −10 mA cm^−2^, 5%Ru‐MoS_2_/CNT requires an overpotential of 50 mV, which is about 141 and 36 mV smaller than that of MoS_2_/CNT and 2%Ru‐MoS_2_/CNT, respectively (Table S2, Supporting Information). The Tafel slopes of both MoS_2_/CNT and Ru‐MoS_2_/CNT are in the range from 40 to 120 mV dec^−1^ (Figure 3b and Figure S10, Supporting Information), suggesting the Volmer–Heyrovsky HER mechanism.^29^ The remarkably small Tafel slope of 5%Ru‐MoS_2_/CNT over that of MoS_2_/CNT suggests superior HER kinetics in 5%Ru‐MoS_2_/CNT.^54^, ^55^ Better HER activity of 5%Ru‐MoS_2_/CNT than MoS_2_/CNT is also reflected by its faster electrode kinetics in view of its smaller charge transfer resistance derived from the electrochemical impedance spectrum (EIS) (Figure 3c). The difference of electrochemically accessible surface area (ECSA) of MoS_2_/CNT, 2%Ru‐MoS_2_/CNT, and 5%Ru‐MoS_2_/CNT electrodes was estimated by determining their double‐layer capacitance (*C*_dl_) by a reported cyclic voltammetry method (Figure S11, Supporting Information).^40^ It can be found that the *C*_dl_ increases as the rise of Ru‐doping amount in Ru‐MoS_2_/CNT (Figure 3d). Considering their structural similarities, the correlation of *C*_dl_ and Ru content in Ru‐MoS_2_/CNT indicates more active sites are generated by Ru‐doping in Ru‐MoS_2_/CNT. However, the slight increase of ECSA of Ru‐MoS_2_/CNT over MoS_2_/CNT cannot fully account for their significantly enhanced HER activities.

**Figure 3:**
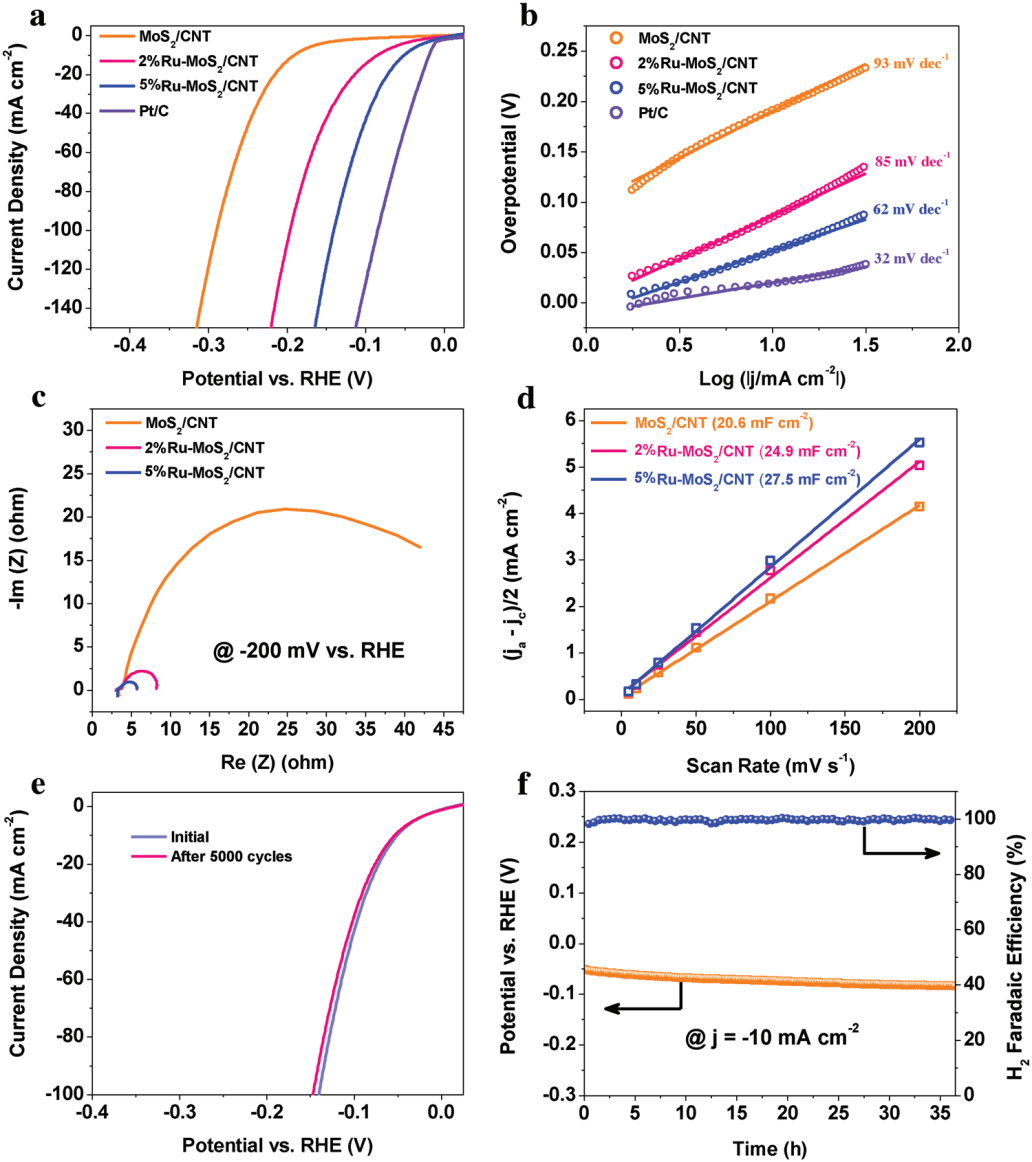
Electrocatalytic HER performance of Ru‐MoS_2_/CNT hybrids. a) Polarization curves, b) Tafel plots, c) EIS spectra, d) extraction of *C*_dl_ by linear fitting of scan rate dependent capacitance currents, e) ADT cyclic stability, and f) choronopotentiometric curve and periodically recorded H_2_ Faradaic efficiencies.

The catalytic durability of 5%Ru‐MoS_2_/CNT was assessed by accelerated degradation test (ADT) and chronopotentiometry. A negligible negative shift of the polarization curve is observed after 5000 continuous cyclic voltammetry sweeps in the potential window between −0.15 and 0.05 V at a scan rate of 100 mV s^−1^ (Figure 3e). Additionally, only a slightly increased overpotential of 23 mV is required to achieve a current density of −10 mA cm^−2^ after continuous operation over 36 h (Figure 3f). The periodically recorded H_2_ Faradaic efficiencies during the chronopotentiometry test are determined to be nearly 100% by online gas chromatograph (Figure 3f). Post‐HER characterizations including TEM, EDS, Raman, and XPS analyses show that the compositions, morphology, and structure of 5%Ru‐MoS_2_/CNT have no observable changes after the chronopotentiometry test (Figures S12 and S13, Supporting Information). These results are indicative of excellent durability of 5%Ru‐MoS_2_/CNT for alkaline HER. Thus, the outstanding electrocatalytic activity and durability enable 5%Ru‐MoS_2_/CNT to be a promising candidate to compete with other HER catalysts for being used in water electrolysis (Table S3, Supporting Information).

Density functional theory (DFT) calculations were performed to deeply understand the roles of Ru‐doping in improving HER activity of Ru‐MoS_2_/CNT. In our theoretical model, a 4 × 4 unit cell of MoS_2_ with Ru‐doping density θ = 1/16 adopts a representative basal plane configuration with isolated Ru doping atom. The calculated total density of states (DOS) of MoS_2_ reveal a bandgap of 1.21 eV, which is consistent with previously reported theoretical and experimental results (**Figure** **4**a).^22^, ^56^ The Fermi level of Ru‐MoS_2_ moves closer to the conduction band, indicative of a n‐type Ru doping. Additionally, some new gap states appear around the Fermi level in Ru‐MoS_2_. These hybridized electronic states have been revealed to be responsible for enhanced hydrogen binding at the in‐plane S sites.^18^, ^22^ For alkaline HER, it has been reported that the energy barriers for water adsorption and dissociation as well as hydrogen binding energy all strongly correlate with HER activity on catalyst surface.^57^, ^58^, ^59^ As shown in Figure 4b, Ru‐MoS_2_ shows much smaller water adsorption energy change than that of pristine MoS_2_, indicating that water molecules are more easily adsorbed on Ru‐MoS_2_ surface to afford the proton source for HER.^59^ After the theoretical identification that in‐plane S sites are the active centers, our calculation results show that water dissociation energy barrier is remarkably lowered at the in‐plane S sites neighboring to the doped Ru atoms in Ru‐MoS_2_ (Figure S14, Supporting Information). Moreover, the hydrogen adsorption free energy (Δ*G*_H_) on the Ru‐bonded in‐plane S sites is strikingly reduced to about 0.19 eV, which is very close to thermoneutral value of Δ*G*_H_ (Figure 4c,d). According to Sabatier principle, a moderate Δ*G*_H_ is conducive to the balance of hydrogen adsorption and desorption, and thus improving HER thermodynamics and kinetics.^6^ The superiority of the improved Δ*G*_H_ on Ru‐MoS_2_ has also been demonstrated by significantly enhanced acidic HER activity of Ru‐MoS_2_/CNT (Figure S15, Supporting Information). Thus, the theoretical results reveal that Ru‐doping can efficiently activate the inert basal plane S sites in MoS_2_ for HER by synergistically improving the water adsorption, dissociation, and hydrogen adsorption/desorption.

**Figure 4:**
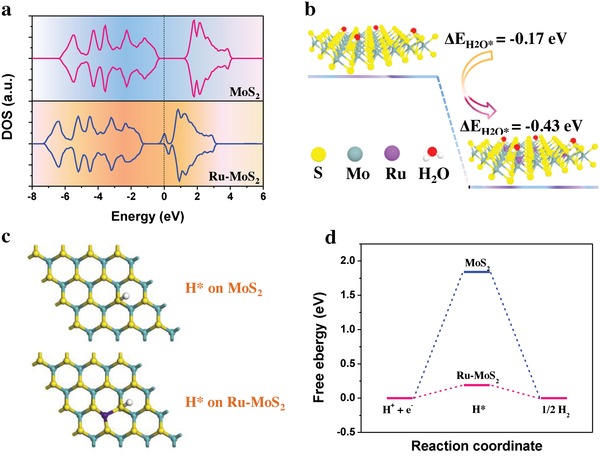
Electronic effects of Ru‐doping on HER activity of Ru‐MoS_2_. a) Total DOS. b) Calculated water adsorption energy change. c) Optimized adsorption configuration of atomic hydrogen. d) Free energy diagram for HER. The calculated data of MoS_2_ were presented for comparison.

In summary, we have designed and synthesized a novel core/shell structured Ru‐MoS_2_/CNT catalyst and demonstrated its superior catalytic performance toward HER. In combination with theory and experiment, we proved that Ru‐doping into basal planes of MoS_2_ is feasible and is an effective methodology for activating the S atoms of inert 2H‐MoS_2_ basal planes for HER. The multiscale electronic and structural engineering strategy developed in this work will open up many new opportunities in exploring cost‐effective electrocatalysts based on transition metal dichalcogenides for practical applications.

## Conflict of Interest

The authors declare no conflict of interest.

## Supporting information

Supplementary
