# Supplementary material for: Engineering MoS2 Basal Planes for Hydrogen Evolution via Synergistic Ruthenium Doping and Nanocarbon Hybridization
Source: Adv Sci (Weinh). 2019 Mar 20;6(10):1900090. doi: 10.1002/advs.201900090 (PMC6523370; doi:10.1002/advs.201900090)
Supplement: Supplementary file 1 — Supplementary [file ADVS-6-1900090-s001.pdf]

Copyright WILEY-VCH Verlag GmbH & Co. KGaA, 69469 Weinheim, Germany, 2019.

ADVANCED SCIENCE
Open Access

Supporting Information

for *Adv. Sci.,* DOI: 10.1002/advs.201900090

Engineering MoS_2 Basal Planes for Hydrogen Evolution via Synergistic Ruthenium Doping and Nanocarbon Hybridization

Xing Zhang, Feng Zhou, Shen Zhang, Yongye Liang, and Ruihu Wang**

Copyright WILEY-VCH Verlag GmbH & Co. KGaA, 69469 Weinheim, Germany, 2019.

Supporting Information

Engineering MoS_2 Basal Planes for Hydrogen Evolution via Synergistic Ruthenium Doping and Nanocarbon Hybridization

Xing Zhang, Feng Zhou, Shen Zhang, Yongye Liang* and Ruihu Wang*

X. Zhang, F. Zhou, S. Zhang Prof. R. Wang

State Key Laboratory of Structural Chemistry, Fujian Institute of Research on the Structure of Matter, Chinese Academy of Sciences, Fuzhou, Fujian 350002, China

E-mail: ruihu@fjirsm.ac.cn

X. Zhang, Prof. Y. Liang

Department of Materials Science and Engineering, Southern University of Science and Technology, Shenzhen 518055, China

E-mail: Liangyy@sustc.edu.cn

X. Zhang, F. Zhou

University of Chinese Academy of Sciences, Beijing 100049, China

Experimental Section

Synthesis of Ru-MoS_2/CNT hybrids. Mildly oxidized multi-walled CNT was prepared according to reported method in literature.[S1] After mildly oxidized multi-walled CNT (20 mg) was dispersed in absolute ethanol (50 mL) by ultrasonication, calculated volumes of 0.1 M $MoCl_5$ and 0.1 M $RuCl_3$ ethanol solution were added with the total amount of metal ions to be 0.2 mmol. Then, NH_4HCO_3 (160 mg) was added and the resultant mixture was magnetically stirred at 800 rpm for 5 h. Solid product was collected by centrifuging and lyophilization. 30 mg of the solid product in one quartz boat was placed at the centre of a 2 inch diameter quartz tube in a single-zone 40 inch horizontal tube furnace. Sulfur powder (320 mg) in another quartz boat was placed in the upstream region of the quartz tube at a distance of 10 cm away from the solid precursor. After flushed with high-purity N_2, the tube furnace was heated from room temperature to 700 °C with a ramping rate of 5 °C min^{-1} and maintained at this temperature for 1 h. During the heating treatment, the ventilation inlet of the quartz tube was shut off while the outlet was kept open and extended by a rubber hose (100 cm in length) whose termination was immersed in water (10 cm below water surface). N_2 flows continuously through the quartz tube (100 sccm) during natural cooling.

Materials characterization. TEM/STEM imaging, electron diffraction and EDS spectroscopy analyses were conducted on an FEI Tecnai G2 F30 microscope operated at an accelerated voltage of 300 kV. XRD measurements were performed on a Rigaku MiniFlex 600 diffractometer using Cu K_α radiation. XPS measurements were performed on a ESCALAB 250Xi spectrometer with a monochromatic Al K_α X-ray source. Raman spectra were taken with Horiba Labram HR800 Evolution spectrometer with a 532 nm excitation laser. Elemental quantitative analysis was performed by inductively coupled plasma optical emission spectrometer (ICP-OES) on a Horiba Jobin Yvon Ultima2 spectrometer.

Electrochemical measurements. The catalyst inks were prepared by dispersing catalysts (10 mg) into a dispersant composed of 50 μl 5 wt% Nafion solution and 950 μl ethanol with the assistance of sonication for ~1 h. The catalyst electrodes were prepared by dropping 50 μl catalyst ink onto a carbon fiber paper (AvCarb MGL190, Fuel Cell Store) to cover an area of 0.5 cm^{-2} and naturally dried at room temperature over 1 h. All electrochemical tests were

taken on a CHI 760E electrochemistry workstation with a coupled three-electrode system. A saturated calomel electrode (SCE) and a graphite rod were used as the reference electrode and counter electrode, respectively. For all of the electrochemical measurements, the corresponding electrolytes were saturated with H_2 by continuous purging with high-purity H_2 (99.999%) during the entire measurement processes. All polarization curves were recorded at a scan rate of 5 mV s^{-1} unless being specifically indicated. All the potentials reported in this work were manually iR-corrected and converted to RHE scale. H_2 Faradaic efficiencies were determined using our previously reported method.[S2]

Theoretical calculations. All calculations on the total energies and atomic structures of the chosen systems were conducted using spin-polarized density functional theory (DFT) with generalized gradient approximation (GGA) for exchange-correlation potential embedded in the Vienna Ab Initio Simulation Package (VASP).[S3-S5] The ion-electron interaction was described with projector augmented wave (PAW) method.[S6] The cutoff energy of the plane-wave basis set was set at 500 eV. In order to avoid interaction between periodic MoS_2 layers, a vacuum space of 15 Å along the z-direction was applied. The Brillouin zoneintegrations were performed by using Monkhorst-Pack 3×3×1 forgeometric optimization. The convergence thresholds for structural optimization was set at 0.01 eV/Å in force.[S7] The convergence criterion for energy is 10^{-5} eV. The van der Waals (vdW) dispersion by employing the D3 method of Grimme was considered for all the calculations.[S8] The climbing image nudged elastic band (CI-NEB) method was used to search the transition states and six images inserted in between two stable states.[S9]

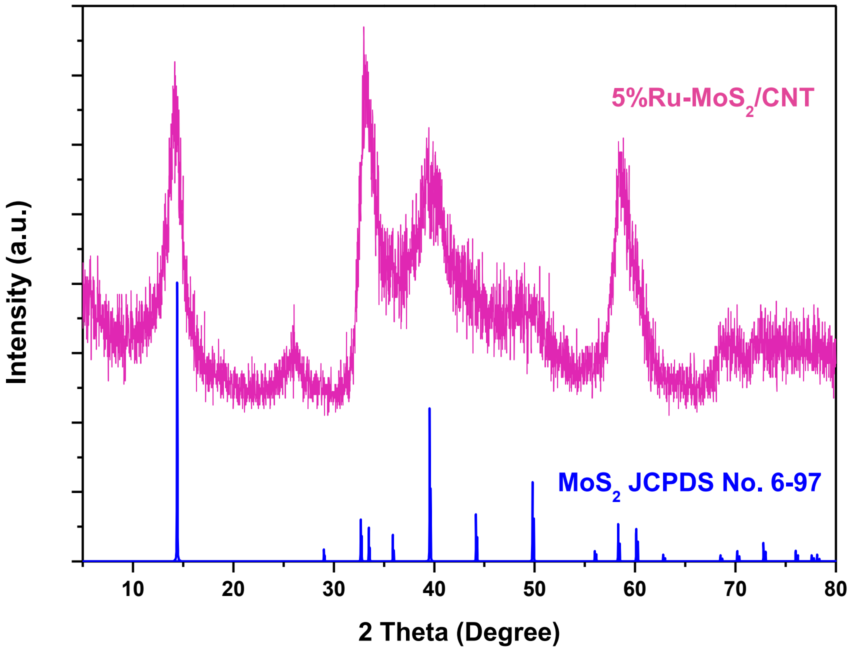

Figure S1. XRD pattern of 5%Ru-MoS_2/CNT.

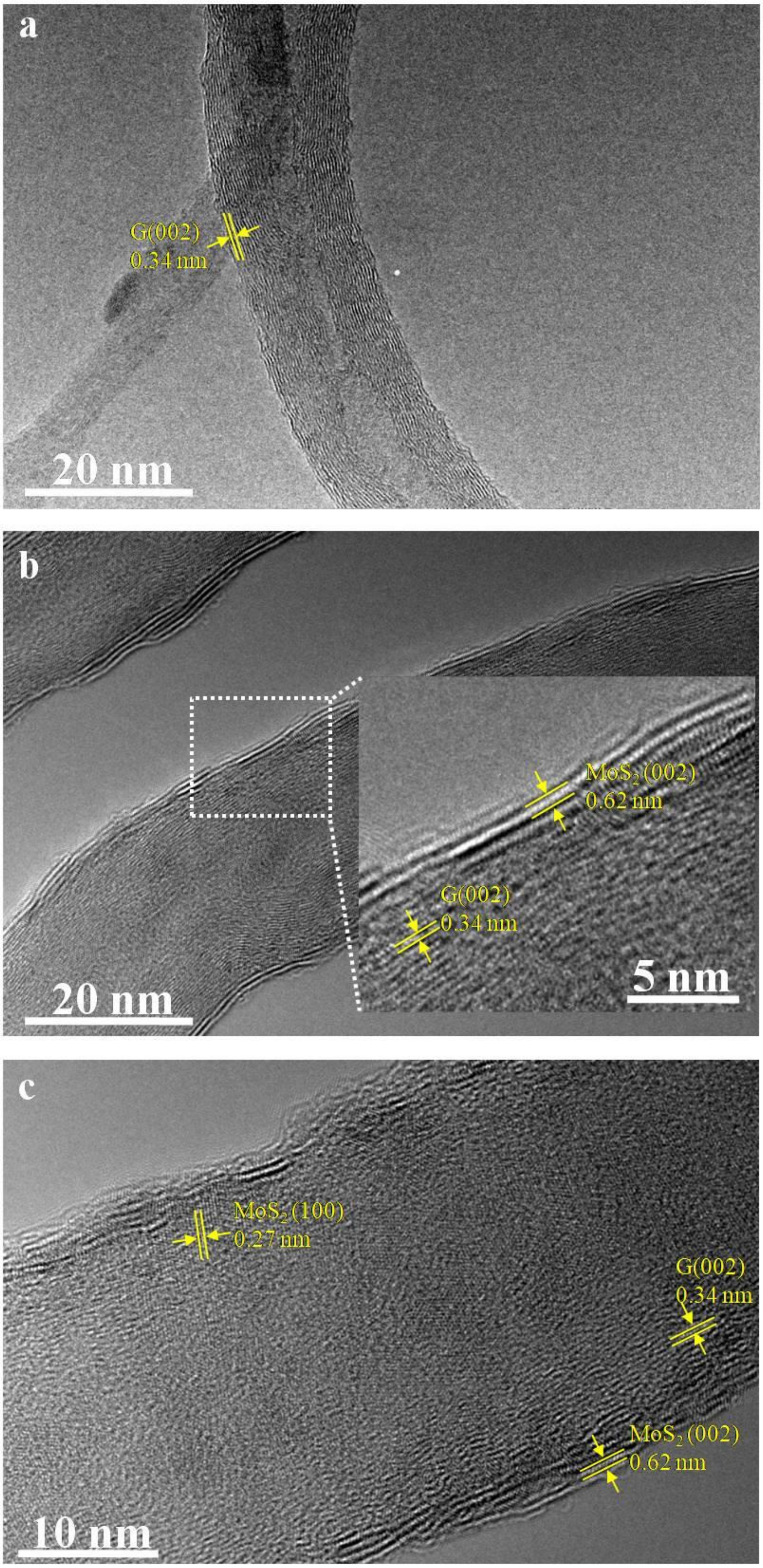

Figure S2. High-resolution TEM images of (a) bare CNT and (b, c) 5%Ru-MoS_2/CNT.

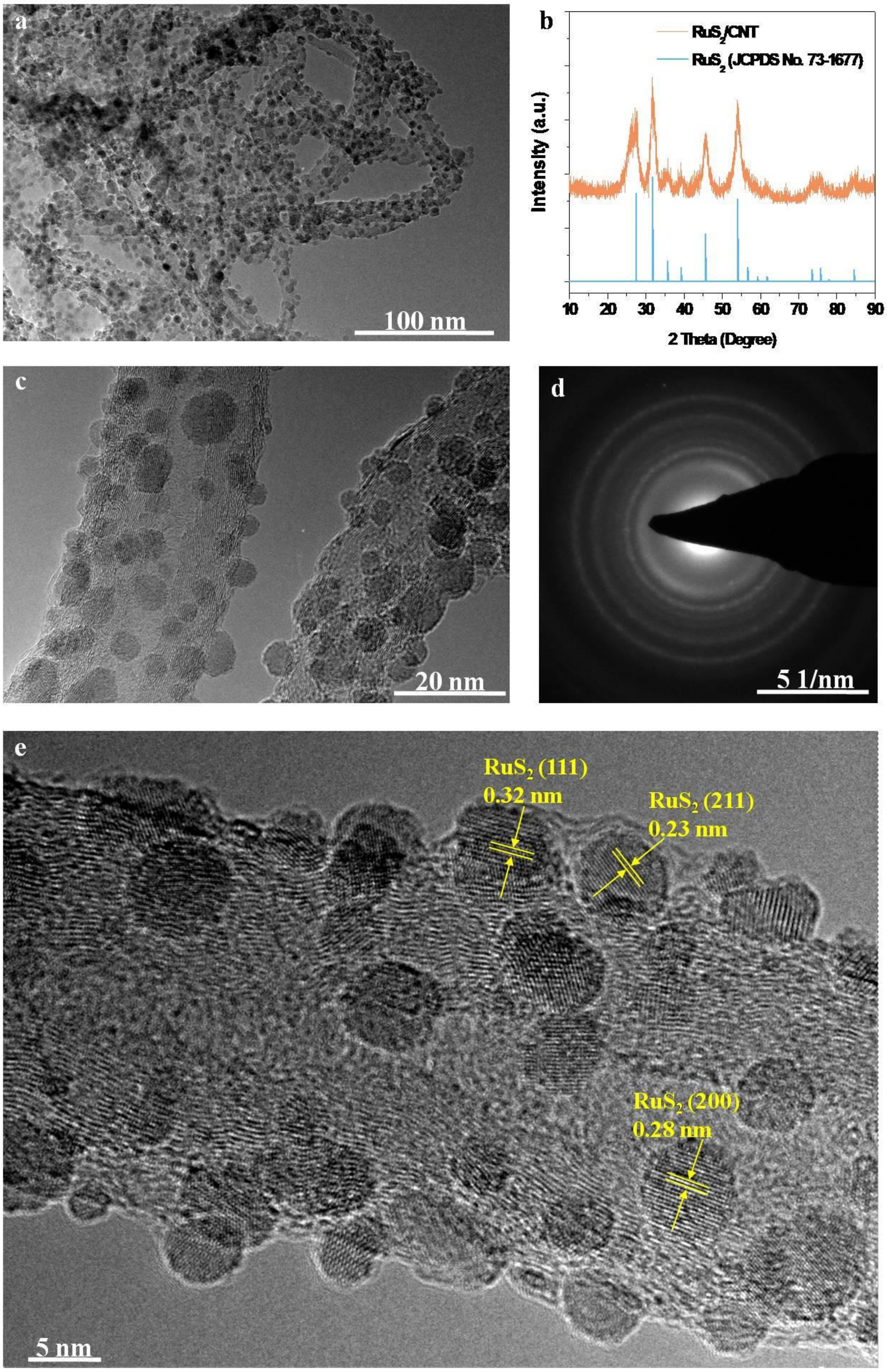

Figure S3. (a, c, e) TEM images, (b) XRD pattern and (d) selective area electron diffraction (SAED) pattern of RuS_2/CNT. The content of RuS_2 was determined to be ~43 wt % by ICP-OES.

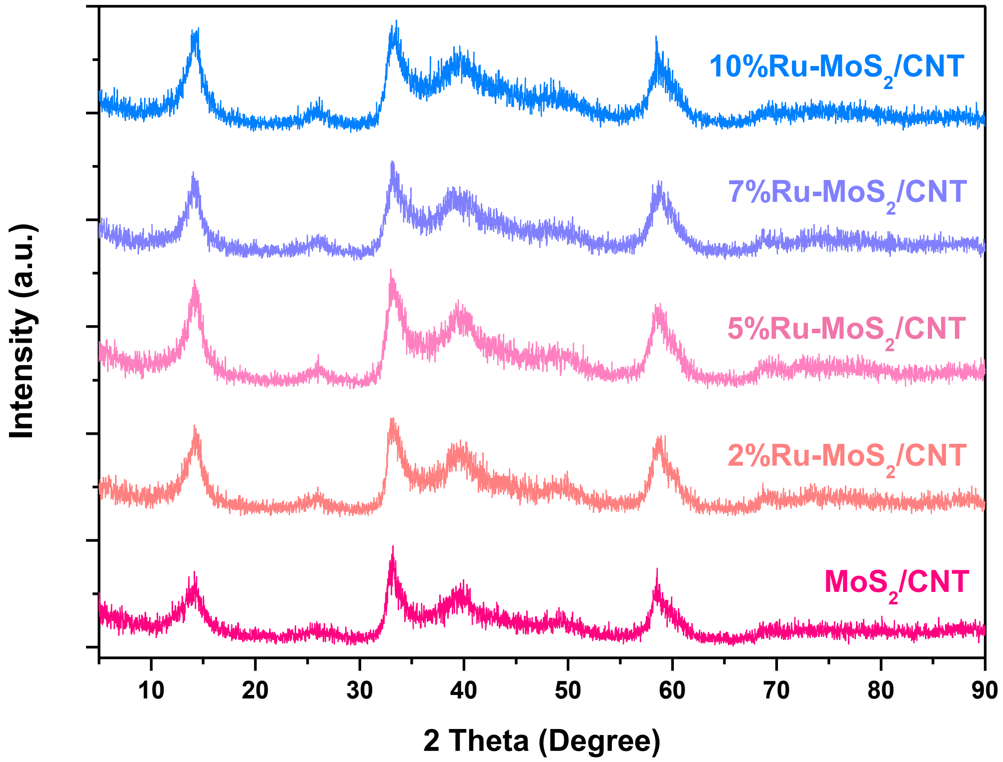

Figure S4. XRD patterns of Ru-MoS_2/CNT with different Ru-doping amount.

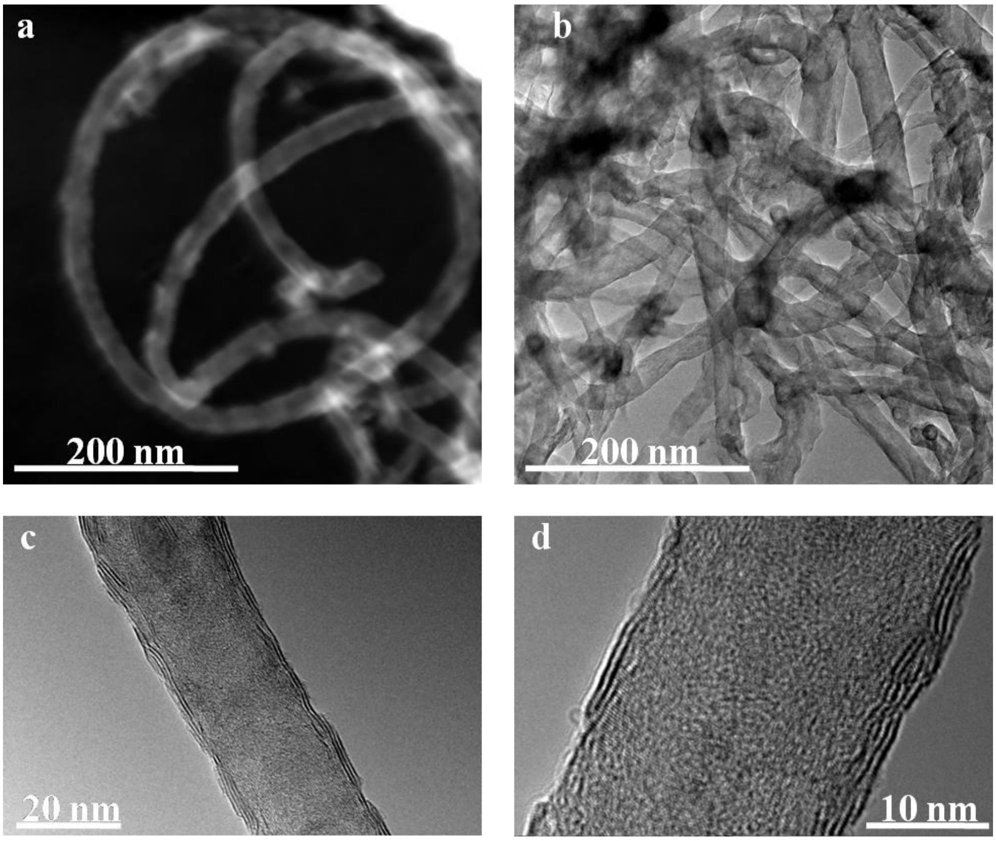

Figure S5. (a) STEM and (b-d) TEM images of 10%Ru-MoS_2/CNT.

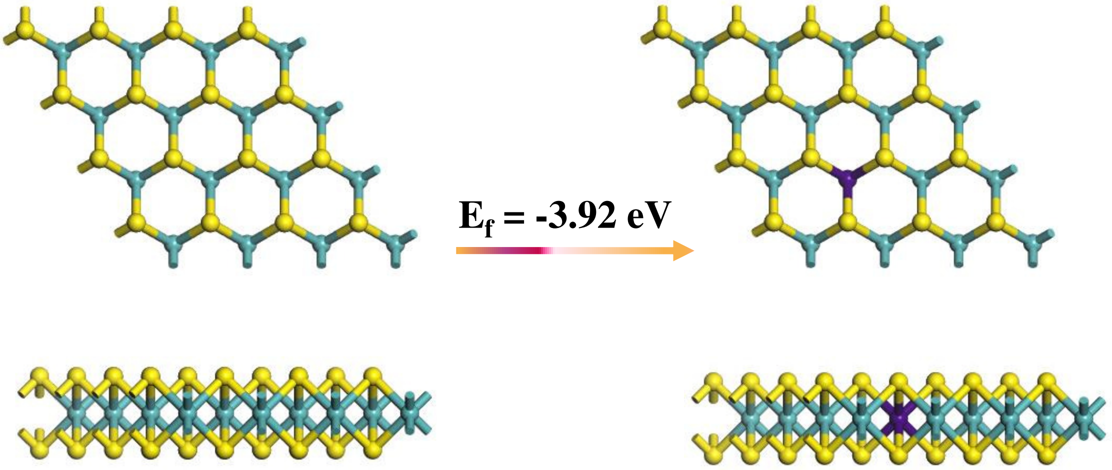

Figure S6. Optimized configuration of Ru atoms in Ru-MoS_2. The formation energy (E_f) for Ru substituting Mo is calculated to be -3.92 eV, suggesting high stability of Ru dopants in MoS_2.

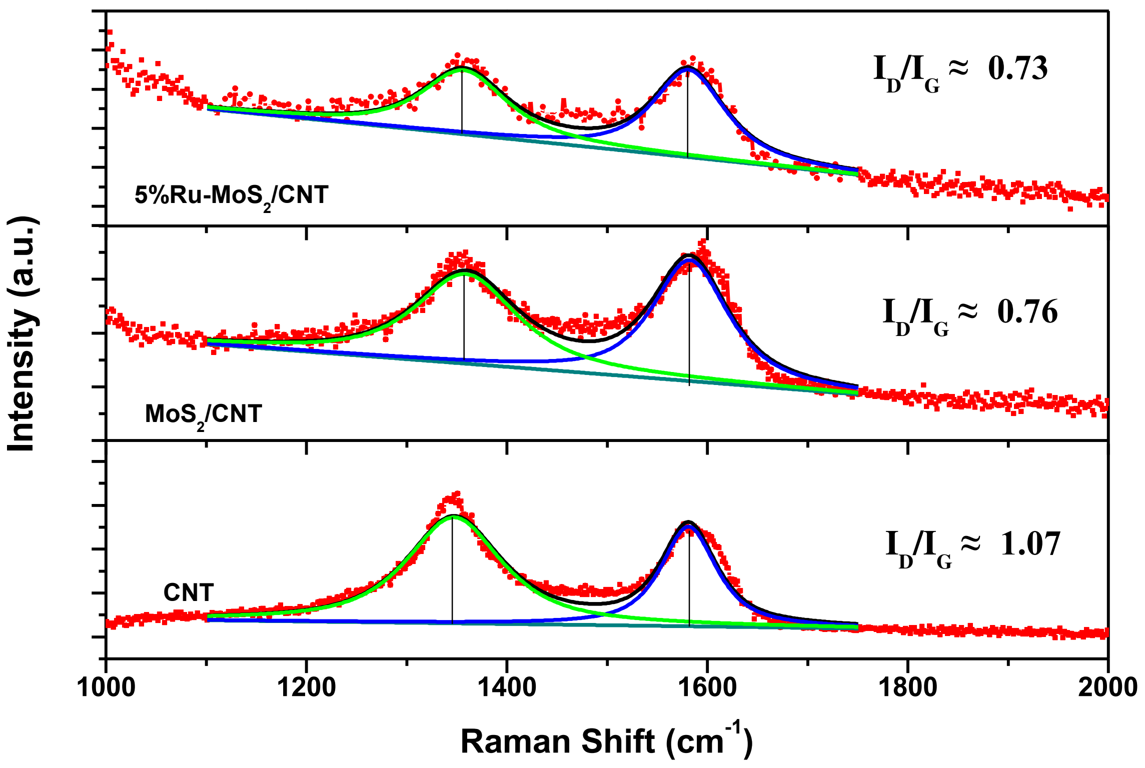

Figure S7. Diconvoluted Raman spectra of CNT, MoS_2/CNT and 5%Ru-MoS_2/CNT for extraction of the intensity ratio of D to G (i.e. I_D/I_G).

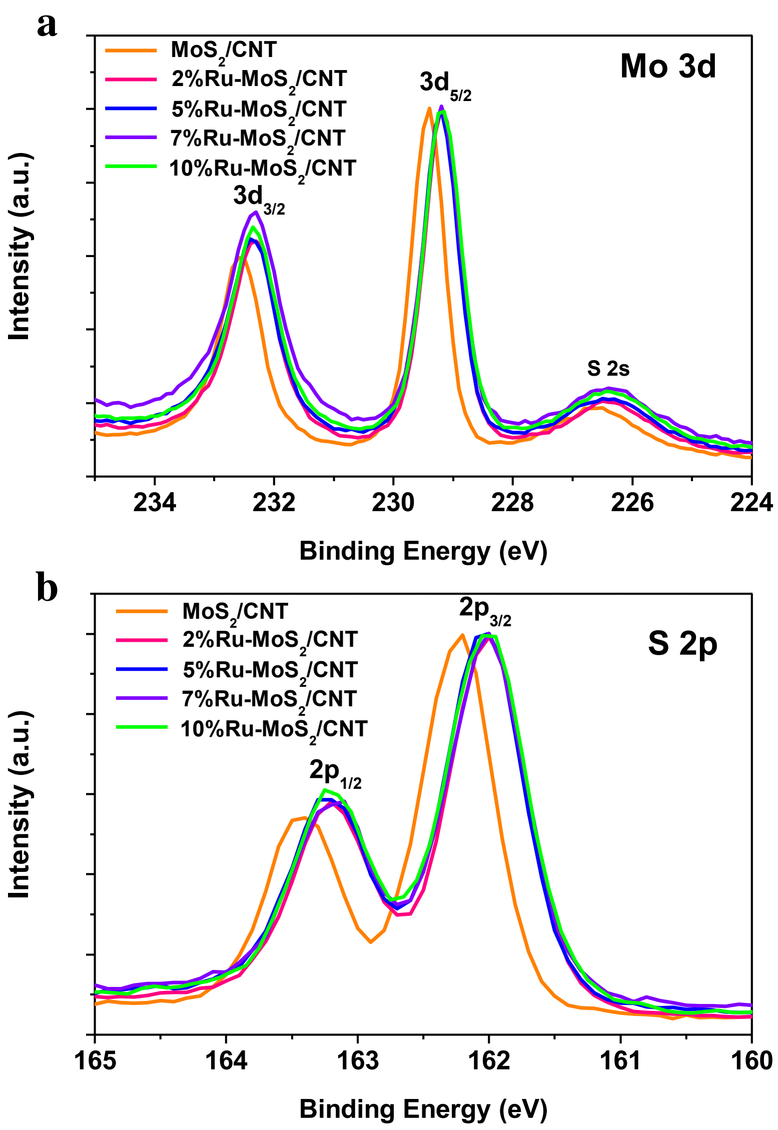

Figure S8. Normalized Mo 3d and S 2p XPS spectra of Ru-MoS_2/CNT with different Ru-doping amount.

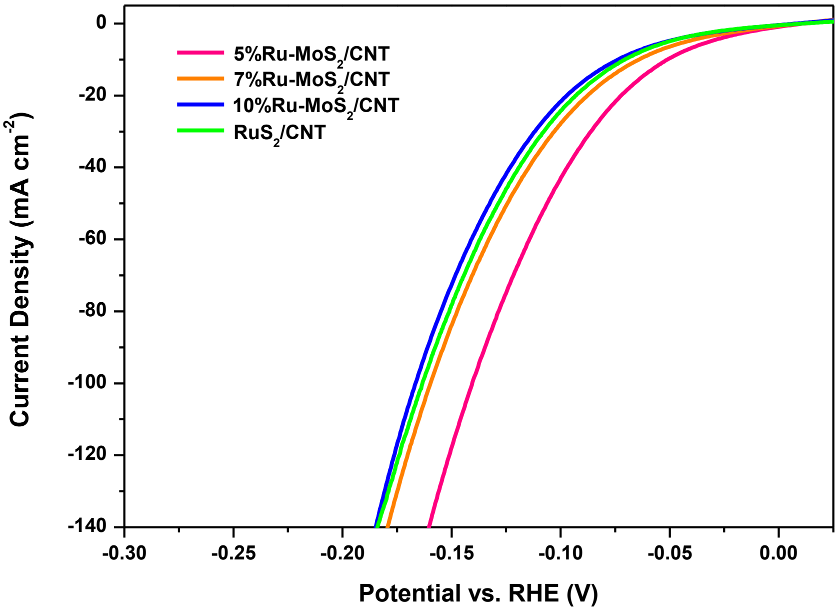

Figure S9. Comparison of HER activities of 7%Ru-MoS_2/CNT, 10%Ru-MoS_2/CNT and RuS_2/CNT with that of 5%Ru-MoS_2/CNT. Ru content in RuS_2/CNT is determined to be nearly eight times larger than that in 5%Ru-MoS_2/CNT.

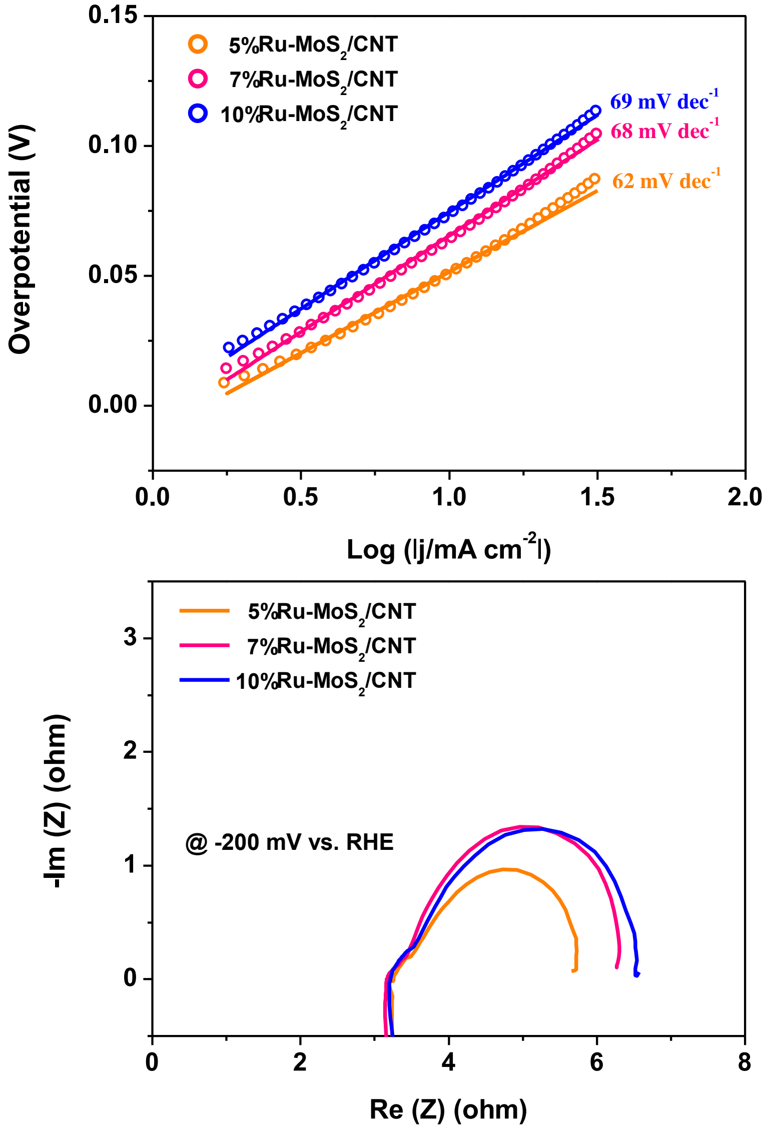

Figure S10. (a) Tafel plots and (b) EIS spectra of 5%Ru-MoS_2/CNT, 7%Ru-MoS_2/CNT and 10%Ru-MoS_2/CNT. The EIS spectra were recorded at biased potential of -200 mV vs. RHE.

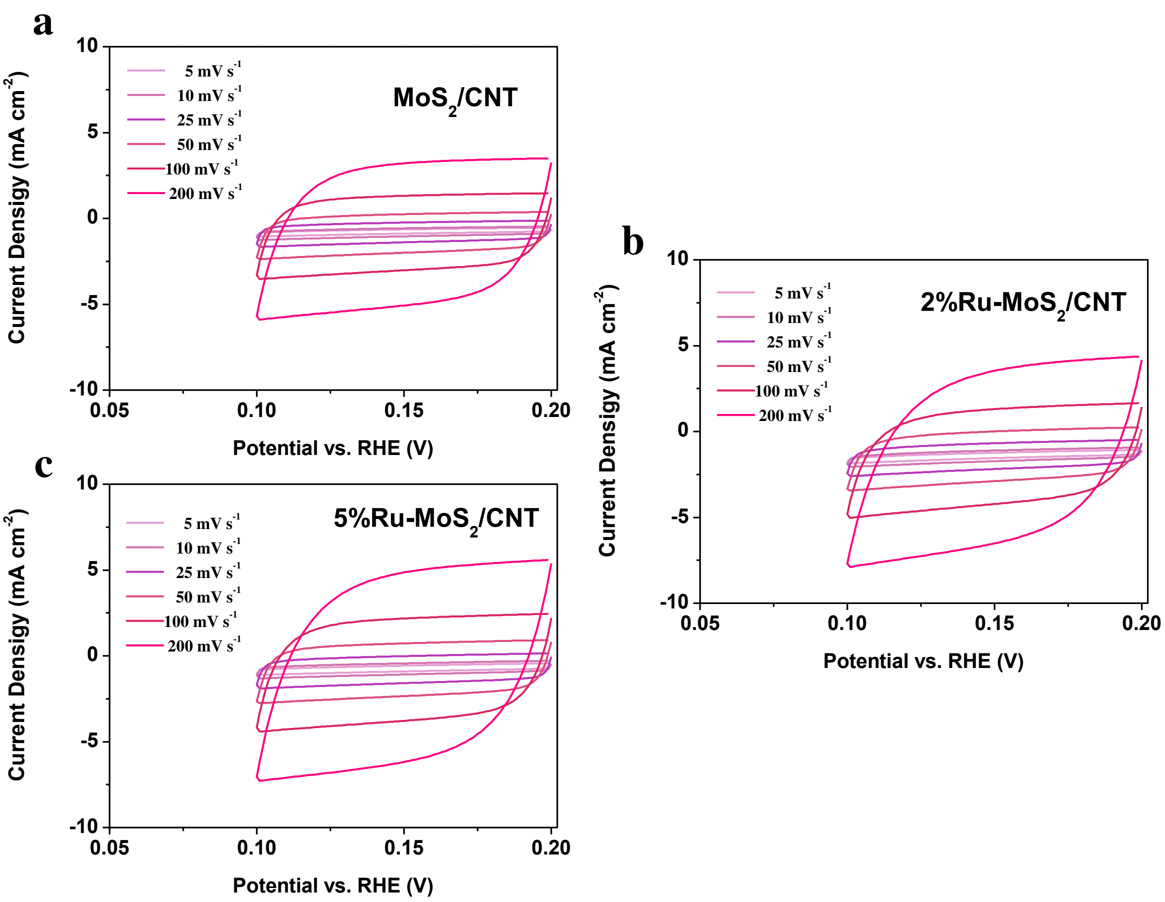

Figure S11. Cyclic voltammograms recorded at different scan rate in the non-Faradaic potential region (0.1-0.2 V vs. RHE) of (a) MoS_2/CNT, (b) 2%Ru-MoS_2/CNT and (c) 5%Ru-MoS_2/CNT.

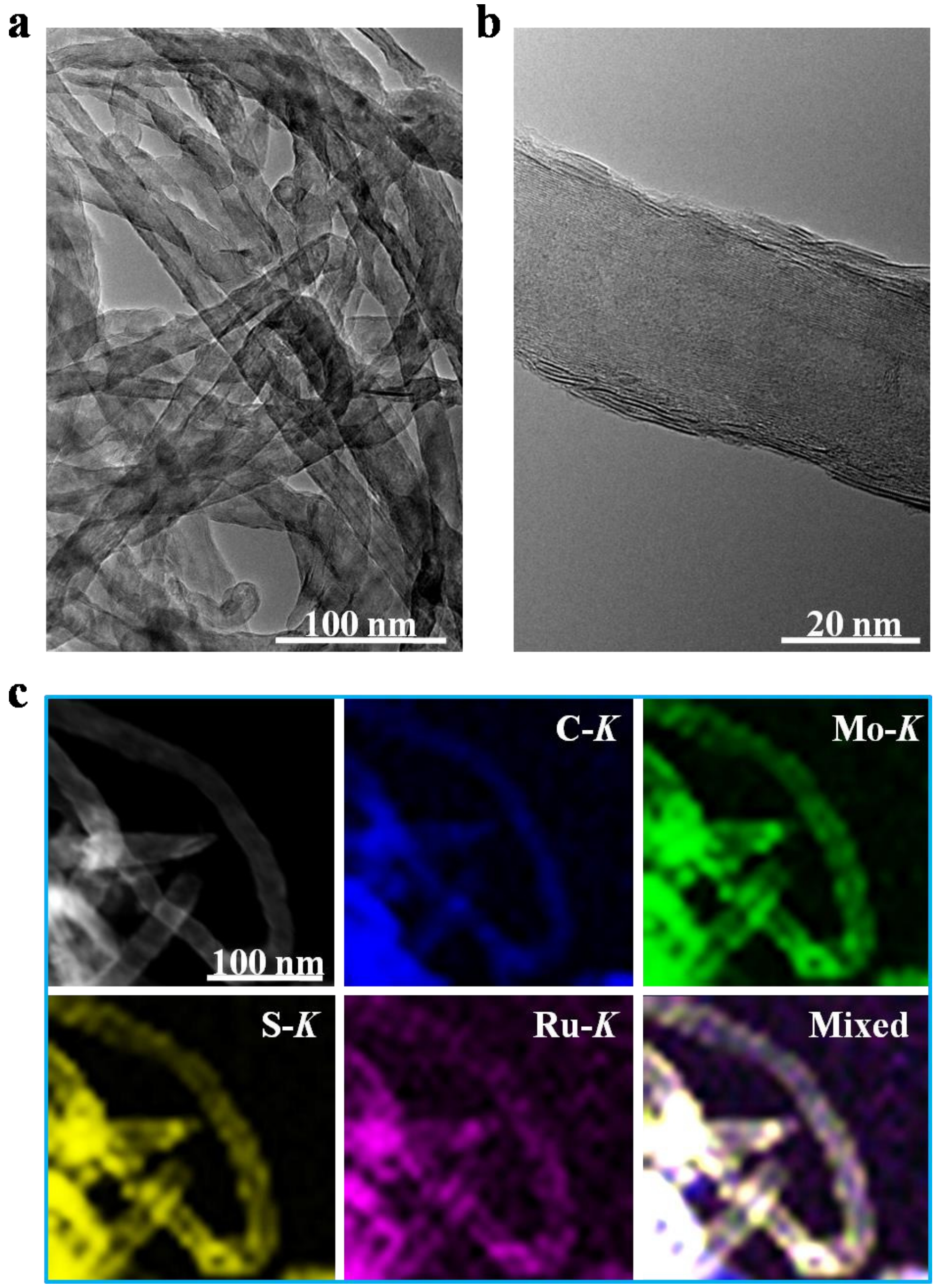

Figure S12. Post-HER characterizations on 5%Ru-MoS_2/CNT after chronopotentiometry test. (a, b) TEM images. (c) STEM and EDS elemental mappings.

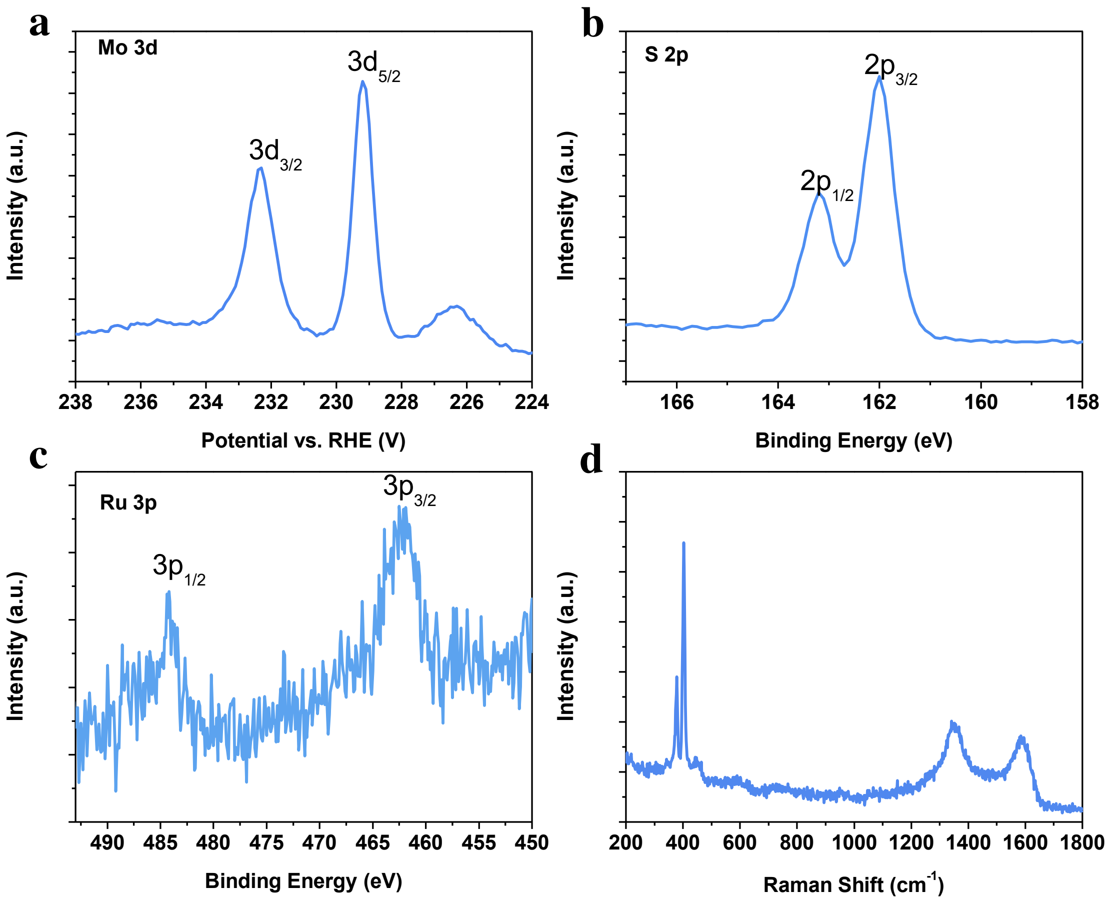

Figure S13. Post-HER characterizations on 5%Ru-MoS_2/CNT after chronopotentiometry test. (a) Mo 3d, (b) S 2p and (c) Ru 3p XPS spectra. (d) Raman spectrum.

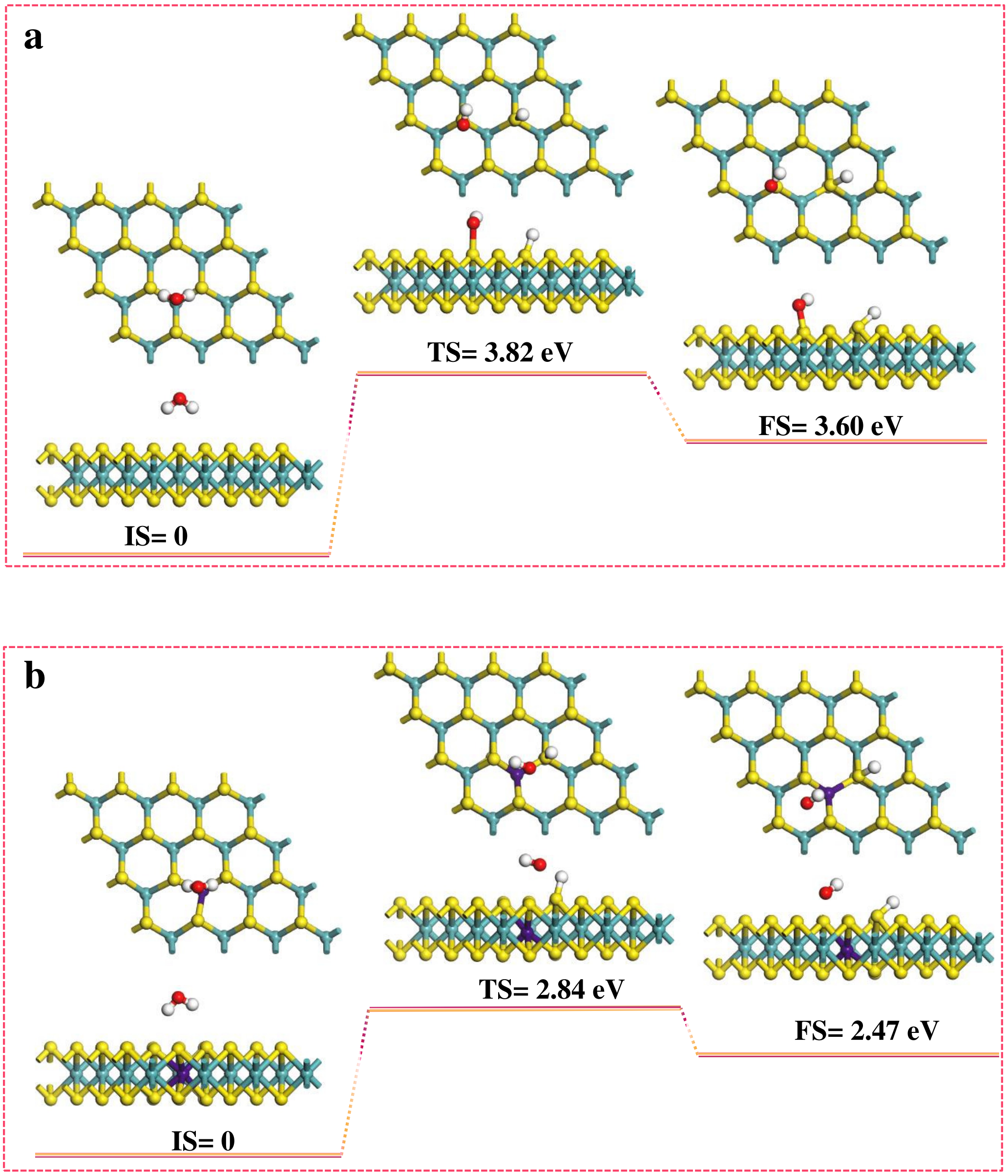

Figure S14. Optimized adsorption configurations and free energy changes in the water dissociation pathways on (a) MoS_2 and (b) Ru-MoS_2.

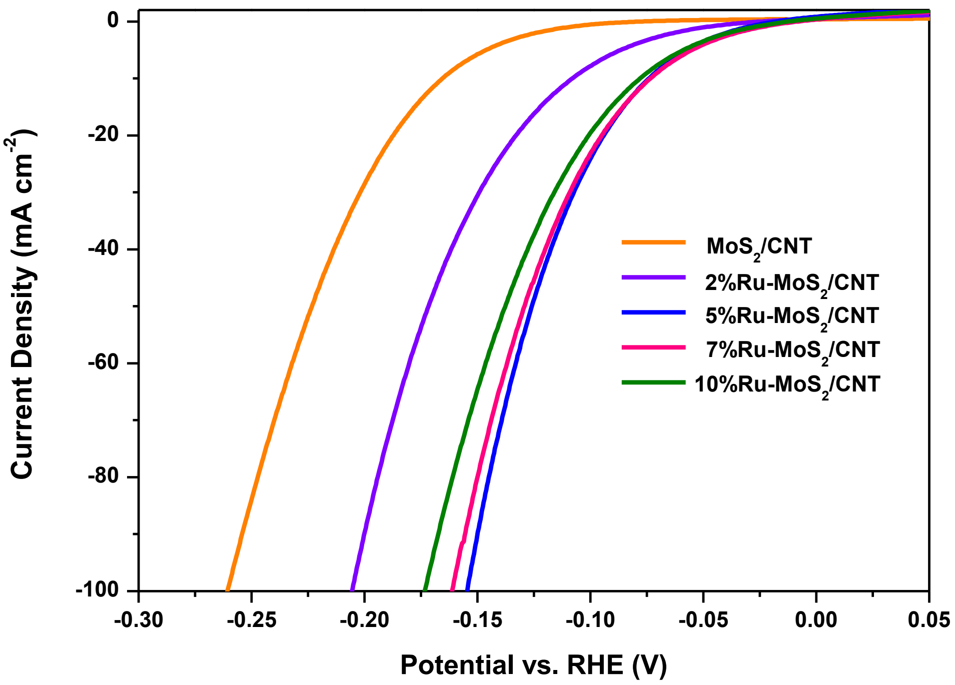

Figure S15. HER activities of MoS_2/CNT and Ru-MoS_2/CNT in 0.5 M H_2SO_4.

Table S1. Contents of Ru in Ru-MoS_2/CNT hybrids determined by ICP-OES.

Initial atomic percent of Ru/(Mo + Ru) in synthesis	Atomic percent determined by ICP-OES	Sample name denoted in this paper
2 at%	2.2 at%	2%Ru-MoS_2/CNT
5 at%	5.2 at%	5%Ru-MoS_2/CNT
7 at%	7.3 at%	7%Ru-MoS_2/CNT
10 at%	10.3 at%	10%Ru-MoS_2/CNT

Table S2. Comparison of HER performance parameters of MoS_2/CNT, Ru-MoS_2/CNT and Pt/C in 1 M KOH.

Catalyst	η_{10} (mV)	Tafel slope (mV dec^{-1})	Exchange current density (mA cm^{-2})
MoS_2/CNT	191	93	0.09
2%Ru-MoS_2/CNT	86	85	0.98
5%Ru-MoS_2/CNT	50	62	1.50
7%Ru-MoS_2/CNT	65	68	1.22
10%Ru-MoS_2/CNT	73	69	1.13
Pt/C	20	32	2.33

Table S3. Comparison of HER activity for high-performance electrocatalysts in alkaline 1 M KOH from recent literatures.

Catalyst	Mass Loading (mg cm^{-2})	η vs. RHE (mV) @ j=10 mA cm^{-2}	Tafel Slope (mV dec^{-1})	Reference (year)
**5%Ru-MoS_2/CNT**	**1.0**	**50**	**62**	**This work**
RuP_2@NPC	1.0	52	69	[S10] (2017)
Ni-Co-P HNBs	2.0	107	46	[S11] (2018)
MoC_x nano-octahedrons	0.8	151	59	[S12] (2015)
Ni-MoS_2	0.89	98	60	[S13] (2016)
Co-MoS_2	0.89	163	158	[S13] (2016)
Fe-MoS_2	0.89	203	181	[S13] (2016)
MoS_2/Ni_3S_2	9.7	118	83	[S14] (2016)
Ni-Mo nanopowder	1.0	~90	N/A	[S15] (2013)
CoP NWAs	0.92	209	129	[S16] (2014)
MoB	2.3	220	59	[S17] (2012)
NiSe NWAs	2.8	96	120	[S18] (2015)
Ni_5P_4 Films	~3.5	150	53	[S19] (2015)
Li-$NiFeO_x$	1.6	88	150	[S20] (2015)
Fe-CoP NWAs	1.03	78	75	[S21] (2017)
$NiCo_2P_x$ NWAs	5.9	58	34	[S22] (2017)
Co-P film	~2.7	94	42	[S23] (2015)
Ni/CeO_2-CNT	0.8	90	N/A	[S24] (2015)

Supplementary References

[S1] Y. Liang, H. Wang, P. Diao, W. Chang, G. Hong, Y. Li, M. Gong, L. Xie, J. Zou, J. Wang, T. Z. Regier, F. Wei, H. Dai, J. Am. Chem. Soc. **2012**, 134, 15849.

[S2] X. Zhang, Y. Liang, Adv. Sci. **2018**, 5, 1700644.

[S3] G. Kresse, J. Furthmüller, Comput. Mater. Sci. **1996**, 6, 15.

[S4] G. Kresse, J. Hafner, Phys. Rev. B, **1993**, 47, 558.

[S5] G. Kresse, D. Joubert, Phys. Rev. B **1999**, 59, 1758.

[S6] H. J. Monkhorst, J. D. Pack, Phys. Rev. B **1976**, 13, 5188.

[S7] S. Grimme, J. Antony, S. Ehrlich, H. Krieg, J. Chem. Phys. **2010**, 132, 154104.

[S8] G. Henkelman, B. P. Uberuaga, H. Jonsson, J. Chem. Phys. **2000**, 113, 9901.

[S9] Q. Tang, D. Jiang, ACS Catal. **2016**, 6, 4953.

[S10] Z. Pu, I. S. Amiinu, Z. Kou, W. Li, S. Mu, Angew. Chem. Int. Ed. **2017**, 56, 11559.

[S11] E. Hu, Y. Feng, J. Nai, D. Zhao, Y. Hu, X. W. Lou, Energy Environ. Sci. **2018**, 11, 872.

[S12] H. B. Wu, B. Y. Xia, L. Yu, X.-Y. Yu, X. W. D. Lou, Nat. Commun. **2015**, 6, 6512.

[S13] J. Zhang, T. Wang, P. Liu, S. Liu, R. Dong, X. Zhuang, M. Chen, X. Feng, Energy Environ. Sci. **2016**, 9, 2789.

[S14] J. Zhang, T. Wang, D. Pohl, B. Rellinghaus, R. Dong, S. Liu, X. Zhuang, X. Feng, Angew. Chem. Int. Ed. **2016**, 55, 6702.

[S15] J. R. McKone, B. F. Sadtler, C. A. Werlang, N. S. Lewis, H. B. Gray, ACS Catal. **2013**, 3, 166.

[S16] J. Tian, Q. Liu, A. M. Asiri, X. Sun, J. Am. Chem. Soc. **2014**, 136, 7587.

[S17] H. Vrubel, X. Hu, Angew. Chem. Int. Ed. **2012**, 51, 12703.

[S18] C. Tang, N. Cheng, Z. Pu, W. Xing, X. Sun, Angew. Chem. Int. Ed. **2015**, 54, 9351.

[S19] M. Ledendecker, S. Krick Calderón, C. Papp, H. P. Steinrück, M. Antonietti, M. Shalom, Angew. Chem. Int. Ed. **2015**, 54, 12361.

[S20] H. Wang, H.-W. Lee, Y. Deng, Z. Lu, P.-C. Hsu, Y. Liu, D. Lin, Y. Cui, Nat. Commun. **2015**, 6, 7261.

[S21] C. Tang, R. Zhang, W. Lu, L. He, X. Jiang, A. M. Asiri, X. Sun, Adv. Mater. **2017**, 29, 1602441.

[S22] R. Zhang, X. Wang, S. Yu, T. Wen, X. Zhu, F. Yang, X. Sun, X. Wang, W. Hu, Adv. Mater. **2017**, 29, 1605502.

[S23] N. Jiang, B. You, M. Sheng, Y. Sun, Angew. Chem. Int. Ed. **2015**, 54, 6251.

[S24] Z. Weng, W. Liu, L.-C. Yin, R. Fang, M. Li, E. I. Altman, Q. Fan, F. Li, H.-M. Cheng, H. Wang, Nano Lett. **2015**, 15, 7704.
